# 2-Deoxy-d-ribose-5-phosphate aldolase (DERA): applications and modifications

**DOI:** 10.1007/s00253-018-9392-8

**Published:** 2018-10-03

**Authors:** Meera Haridas, Eman M. M. Abdelraheem, Ulf Hanefeld

**Affiliations:** 1 Biokatalyse, Afdeling Biotechnologie, Technische Universiteit Delft, Van der Maasweg 9, 2629 HZ Delft, The Netherlands; 2 Chemistry Department, Faculty of Science, Sohag University, Sohag, 82524 Egypt

**Keywords:** Aldol reaction, Aldolase, DERA, C–C bond, Protein engineering, Immobilization

## Abstract

2-Deoxy-d-ribose-5-phosphate aldolase (DERA) is a class I aldolase that offers access to several building blocks for organic synthesis. It catalyzes the stereoselective C–C bond formation between acetaldehyde and numerous other aldehydes. However, the practical application of DERA as a biocatalyst is limited by its poor tolerance towards industrially relevant concentrations of aldehydes, in particular acetaldehyde. Therefore, the development of proper experimental conditions, including protein engineering and/or immobilization on appropriate supports, is required. The present review is aimed to provide a brief overview of DERA, its history, and progress made in understanding the functioning of the enzyme. Furthermore, the current understanding regarding aldehyde resistance of DERA and the various optimizations carried out to modify this property are discussed.

## Introduction

**Scheme 1:**
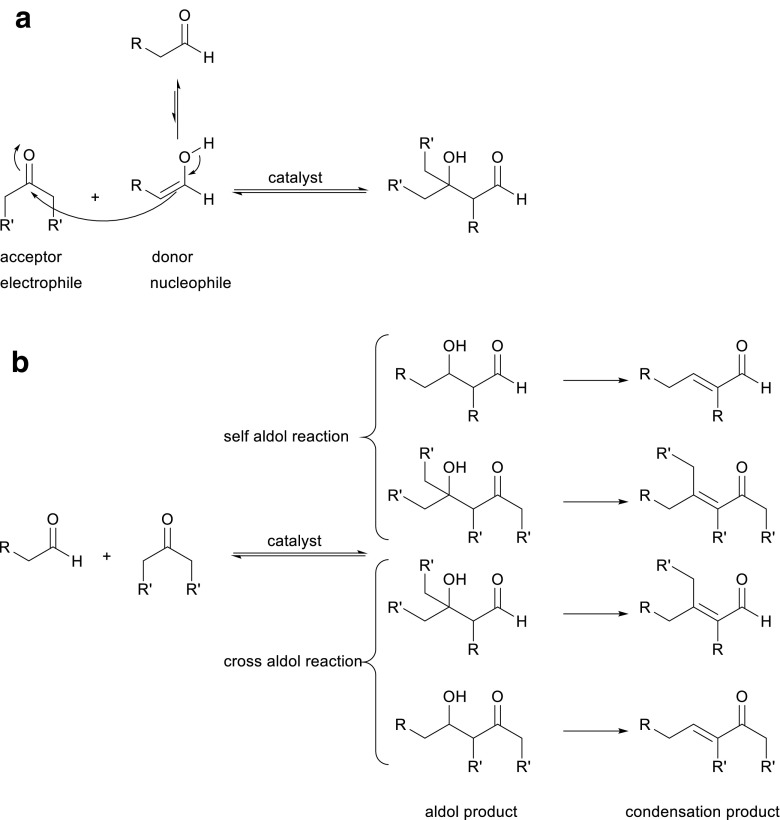
**a**, **b** The aldol reaction is very versatile but at the same time, a lack of control of acceptor and donor as well as the reaction conditions can lead to a mixture of products

The aldol reaction is an important C–C bond forming reaction in organic chemistry that provides access to the aldol motif (Alcaide and Almendros 2003; Li 2005; Sukumaran and Hanefeld 2005). It is the reaction of an enolizable aldehyde or ketone that acts as nucleophile (also called donor) with a second aldehyde or ketone that functions as electrophile and is also called acceptor (Scheme 1a) (Mestres 2004; Li 2005; Mlynarski and Paradowska 2008; Clayden et al. 2012; Müller et al. 2013, 2015). There are two key problems in the aldol reaction, both associated with selectivity (Scheme 1). The first is substrate selectivity, i.e., which of the two aldehydes or ketones acts as donor and which as acceptor, ensuring that the right product is formed. Two self-aldol reactions can occur and two cross-aldol reactions might take place, but only one reaction is the desired reaction. The second selectivity-related problem is that the aldol reaction is very readily followed by a condensation step that leads to the elimination of water and formation of undesired products. Enzymes, i.e., aldolases, efficiently select only one donor and fix which cross-aldol reaction occurs, suppressing self-aldol reactions. Moreover, they ensure that no condensation takes place; consequently, only a single product is obtained and no selectivity problems arise. The aldol reaction is critical in the metabolic context and is an important biochemical process for the production of naturally occurring carbohydrates (Clapés 2015). Recently, improved variants of the aldol reaction, including the enzymatic reaction, have been developed. Enzymatic aldol reactions are attractive for the synthesis of biologically important organic compounds, such as carbohydrates and amino acids (Mlynarski and Paradowska 2008; Clapés 2015). Also, the aldol reaction in general and the enzyme-catalyzed variant in specific have been used in the large-scale synthesis of the commodity chemical pentaerythritol and cholesterol-lowering drugs such as atorvastatin (Mestres 2004; Weissermel and Arpe 2003; Patel 2018).

**Scheme 2:**
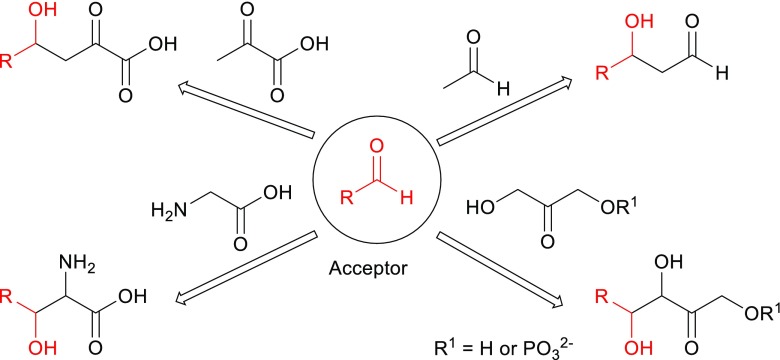
Four groups of aldolases according to the functionality of their donor substrates, above the arrows (acetaldehyde, dihydroxyacetone (phosphate), pyruvate dependent or phosphoenolpyruvate, and glycine)

In addition to the abovementioned selectivities that aldolases introduce into the aldol reaction, they offer a further advantage: They are stereoselective. Overall, aldolases catalyze the reversible stereoselective aldol addition of a donor compound such as a ketone, to an acceptor compound like an aldehyde, yielding the product with high levels of stereocontrol at the newly formed stereogenic centers (Scheme 2). Based on their excellent selectivity for donors, aldolases are grouped according to the four donors that they can utilize. Group I utilizes an activated acetaldehyde as donor and is called acetaldehyde dependent, group II dihydroxyacetone phosphate (DHAP) and dihydroxyacetone dependent, group III pyruvate dependent or phosphoenolpyruvate dependent, and group IV glycine dependent (Clapes and Garrabou 2011; Müller 2012) (Scheme 2). As the number of donors is rather limited, much research is concentrated on finding aldolases with a broader donor scope or aldolases which are specific for yet undescribed donors. A particular success in this area is fructose-6-phosphate aldolase (FSA), an aldolase that accepts dihydroxyacetone as donor but also other ketones and even some aldehydes (Garrabou et al. 2009; Junker et al. 2018). This donor or nucleophile promiscuity was recently reviewed (Hernandez et al. 2018).

Aldolases can also be classified according to the activation mode of the donor carbonyl group. Class I aldolases form a Schiff base between the donor substrate and the NH_2_ group of an active site lysine residue, after which the activated donor stereoselectively adds to the acceptor aldehyde via the enamine intermediate (Scheme 4a). On the other hand, class II aldolases require a divalent metal ion cofactor such as Zn^2+^, Fe^2+^, Mg^2+^, or Co^2+^, which act as a Lewis acid and activate the nucleophile via coordination to the carbonyl group of the donor. Glycine-dependent aldolases work according to yet another mechanism, using pyridoxal phosphate as cofactor (Sukumaran and Hanefeld 2005; Samland and Sprenger 2006; Clapés 2015).

**Scheme 3:**
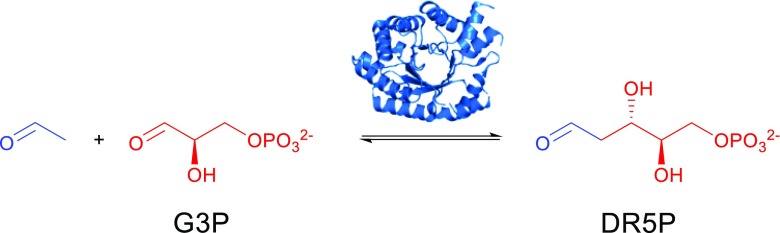
The in vivo 2-deoxy-d-ribose-5-phosphate reaction catalyzed by DERA

Here, in this review, the focus is on 2-deoxy-d-ribose 5-phosphate aldolase (DERA), which is an acetaldehyde-dependent (group I) aldolase. It converts acetaldehyde to yield 2-deoxy aldehydes with a single new stereo center (Scheme 3). First, a brief introduction to the enzyme is provided, followed by its discovery and structure. Then, synthetic applications of DERA and approaches which have been used to improve its catalytic activity and aldehyde tolerance are discussed.

## 2-Deoxy-d-ribose-5-phosphate aldolase

DERA catalyzes the reversible aldol reaction of acetaldehyde and glyceraldehyde-3-phosphate (G3P) to give 2-deoxyribose-5-phosphate (DR5P) (Scheme 3). Interestingly, this enzyme is special among aldolases; in that, it uses an aldehyde rather than a ketone as the natural donor; the only other exception being the promiscuous activity observed for FSA (see above). All known DERAs work according to the class I mechanism. DERA accepts a number of aldehydes with long chains up to four carbon atoms (Clapés 2015) and generates (*S*)-configured stereogenic center in G3P and equivalent stereochemistry in other molecules. Another interesting property of DERA is the fact that it can catalyze sequential aldol reactions yielding 2,4,6-trideoxyhexoses, which can be valuable intermediates for the production of atorvastatin and cholesterol-lowering drugs (Gijsen and Wong 1994; Wong et al. 1995; Patel 2018). The sequential addition of two molecules of acetaldehyde and chloroacetaldehyde was found to give highly stereospecific polyol systems where the stereo selectivity was controlled by the enzyme and not the substrate (Gijsen et al. 1996). This sequential aldol reaction is thermodynamically controlled and stops when a stable intra-molecular hemiacetal is formed.

In 1952, Racker reported that cellular extracts of *E. coli* (EC) catalyze the reversible reaction of G3P with acetaldehyde to give DR5P (Racker 1952). It was later found that the equilibrium of the reaction favors the formation of DR5P, with an equilibrium constant of 4.2 × 10^3^ M^−1^ (Pricer and Horecker 1960). Over the years, DERA has been identified in a wide variety of plant and animal tissues and these have been characterized with respect to several parameters (Table 2). In humans, it is most expressed in lung and liver cells and is involved in stress response by delaying or minimizing stress-induced damage in these cells (Salleron et al. 2014). In 1965, it was demonstrated that one active site is present per monomer of enzyme (Hoffee et al. 1965). DERA is encoded by the *deo*C gene and the sequence of DERA_EC_ was first reported in 1982, when the enzyme was isolated from *E. coli* strain K-12 (Valentin-Hansen et al. 1982). The enzyme is composed of 259 amino acids, with a molecular weight of 27.7 kDa. The amino acid composition of the enzyme isolated from *S. typhimurium* was reported earlier (Hoffee 1968). The enzyme isolated from the two organisms had very similar amino acid compositions and the analysis of results of active-site labelling led to the hypothesis that the lysine residue at position 167 (Lys167) probably forms the Schiff base with the acetaldehyde donor in DERA_EC_ (Hoffee et al. 1974; Valentin-Hansen et al. 1982).

X-ray structures of DERA_EC_ showed that the enzyme exhibits the common TIM (α/β)_8_-barrel fold. It was reported that DERA_EC_ exists as a dimer in crystal, though the functional role of the dimerization in catalysis was unclear at the time (Heine et al. 2004). Crystal structures of DERAs from different organisms were compared and it was observed that a water molecule was conserved in all the crystal structures, indicating its importance in the catalytic mechanism.

Structural studies of the enzyme isolated from the archaeon *Aeropyrum pernix* showed that the enzyme had a stable tetrameric structure, even though the monomeric structure was quite similar to that of DERA_EC_ (Hoffee 1968). On comparing the quaternary structures of *E. coli* and *A. pernix* with that of the thermophilic *Thermus thermophilius* HB8, it was found that the monomeric forms of all three proteins were very similar, unlike their oligomeric forms (Lokanath et al. 2004). Hence, it was concluded that the tetrameric form played an important role in improving the thermal stability of the protein but not the catalytic activity. A recent study on the organization of DERA structures from a range of organisms adapted either to cold or hot environments indicated that flexibility is linked to activity (Dick et al. 2016a).

**Scheme 4:**
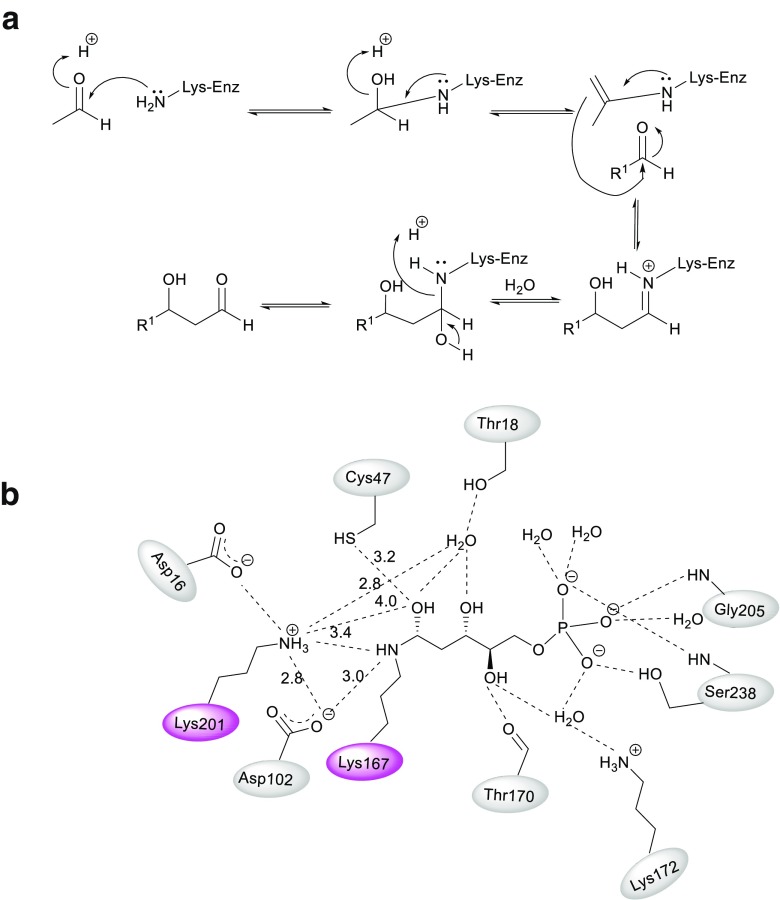
**a** General class I aldolase mechanism shows the role of enamine intermediate in the chemical mechanism. **b** Two-dimensional diagram shows the active side contacts

Crystallographic studies carried out in the early twenty-first century identified a carbinolamine intermediate and a second lysine residue at position 201 (Lys201) that was present quite close to Lys167 Heine et al. 2001). Several mutants were created to study the role of these residues and it was experimentally confirmed that Lys167 and Lys201 were critical for the catalytic activity of DERA. It was reported that while Lys167 is directly involved in the formation of the Schiff base and Lys201 is probably involved in the perturbation of pK_a_ for Lys167 (Scheme 4).

## Substrate specificity

Early reports on DERA were mostly focused on identifying the basic properties of the enzyme and in understanding the natural reaction catalyzed by it. The substrate specificity of the enzyme was first studied in 1960 through which it was concluded that DERA from *Lactobacillus plantarum* (DERA_LP_) was highly specific towards the donor aldehyde (acetaldehyde) and did not utilize propionaldehyde, glycoaldehyde, or pyruvic acid as donor. At the same time, the acceptor aldehyde need not necessarily be glyceraldehyde-3-phosphate and the enzyme also utilized other sugars like d-erythrose as acceptors (Pricer and Horecker 1960). Later this was corrected and propionaldehyde was demonstrated to be a donor for DERA_LP_ (Rosen et al. 1965). Similar studies conducted by Wong et al. at a later time further concluded that acetaldehyde indeed could be replaced by other aldehydes or ketones like acetone and fluoroacetone (Table 1). It was noticed by all studies that non-natural substrates required very large amounts of enzyme to obtain meaningful yields. It was also of particular interest that the bond formation of fluoroacetone was observed exclusively on the non-fluorinated carbon (Barbas et al. 1990). Meanwhile, it was also reported that the enzyme was highly specific towards DR5P and did not cleave ribose–5–phosphate or 2–deoxyribose–1–phosphate (Hoffee 1968).

**Table 1:**
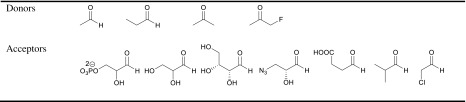
Some reported donors and acceptors (Barbas et al. 1990, Chen et al. 1992, Wong et al. 1995). If stereocenters are drawn without stereoinformation, both diastereoisomers are accepted

Now it is known that DERA accepts a wide range of substrates as the acceptor molecule in aldol condensation reactions. Steric hindrance at the active site was identified as a possible reason for the rejection of several substrates like chloroacetaldehyde and hydroxyacetaldehyde (glycoaldehyde) as donors (Chen et al. 1992). However, these compounds did give aldol products when tested as acceptor substrates, even though the reaction rates were much lower when compared to the natural substrate G3P. In fact, for all acceptors and donors tested, the performance of the enzyme in terms of reaction rate was several folds higher when the natural substrates were used, even though other substrates gave rise to interesting final products. Clearly, even though DERA is the first aldolase to accept both aldehyde and ketone donors, it is still quite restricted towards the size of the donor, and smaller aldehydes are preferred. Such a restriction towards the donor has been observed for other aldolases as well and is the basis of the above mentioned grouping of the aldolases (Scheme 2).

The configuration of the C2 carbon of the acceptor plays an important role in the enzyme action. It was observed that when polar groups were present at this position, the d isomers were preferred over L isomers. In particular, even though the K_M_ of the enzyme towards d and L isomers of G3P were comparable, the reaction with d-G3P proceeded 20 times faster. This was attributed to the favorable orientation of the d-isomer in the active site. However, similar behavior was not observed for stereoisomers of glyceraldehyde, which is smaller in bulk. A reverse enantioselectivity was observed when hydrophobic groups were present at the same position (Liu and Wong 2002).

**Scheme 5:**
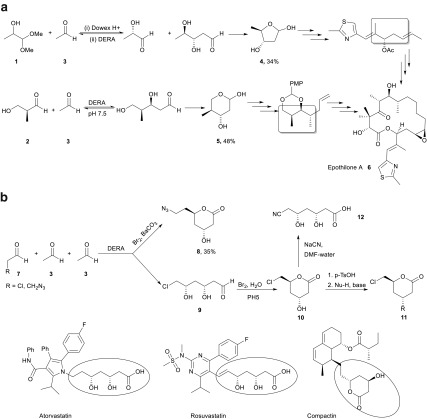
**a** Synthesis of epothilone A using DERA as catalyst due to synthesis of two fragments **4** and **5**. PMP = 4-methoxyphenyl. **b** DERA as a catalyst for the preparation of statin intermediates

Gijsen and Wong reported the first successful DERA-catalyzed sequential aldol reaction and also studied the substrate specificity for such tandem reactions. The sequential addition of three achiral aldehydes in the presence of DERA was reported to give rise to a cyclic system, as shown in Scheme 5b. First, the acetaldehyde donor reacts with a C2-substituted aldehyde (acting as the acceptor) to form a highly stereospecific aldol product, which further reacts with another acetaldehyde molecule to give rise to a stable hemiacetal. It was also reported that DERA did not accept all substituted aldehydes as acceptor substrates in the reaction. In fact, chloroacetaldehyde gave the best yield among the tested acceptor substrates, proving the excellent selectivity of DERA in refusing it as donor (Gijsen and Wong 1994).

Next to all the advantages mentioned, DERA does have disadvantages. The key problem is the very high chemical reactivity of acetaldehyde that is detrimental for proteins in general, and DERA in specific. Therefore, this parameter, acetaldehyde resistance, has been carefully studied for all DERAs described to date (Table 2).

**Table 2:** Overview of enzyme properties and acetaldehyde resistance of DERA isolated from different organisms

Source	Characterization					Acetaldehyde resistance		Reference
	Molecular mass^a^ (kDa)	Optimum pH	Optimum temperature (°C)	K_M_ (mM)	V_max_^b^ or specific activity^c^ (U mg^−1^)^d^	Activity	Reaction conditions	
Rat liver	n.s.	7.5	n.s.	0.17	106.5^c^	n.s.		(Groth 1967)
Environmental DNA libraries	23.9	7	35	0.038	2.9^b^	n.s.		(You et al. 2013)
Mesophiles								
*Salmonella typhimurium*	28.7	7.3–8.4	n.s.	0.1	3500^b^	No quantitative data, but 99% activity loss reported in the presence of acetaldehyde		(Hoffee 1968)
*Steptococcus lactis* subsp*. diacetylactis DRC3*	n.s.	6.8	45	0.6*	n.s.	n.s.		(Lees and Jago 1977)
*Klebsiella pneumoniae*	27.6	7.5	37	n.s.	2.5^c^	n.s.		(Horinouchi et al. 2003)
*Escherichia coli*	28	7.5	n.s.	0.23	58^c^	0% retention of activity	2 h 300 mM 25 °C	(Sakuraba et al. 2007)
*Escherichia coli*	n.s.	n.s.	n.s.	0.29	16^c^	Half-life = 25 min	300 mM 25 °C	(Kullartz and Pietruszka 2012)
*Yersinia* sp*.* EA015	24.8	6	50	9.1	137^c^	Maximal activity at 200 mM		(Kim et al. 2009)
*Paenibacillus* sp*.* EA001	24.5	6	50	145	62^c^	n.s.		(Kim et al. 2010)
*Rhodococcus erythropolis*	22.9	7	25^e^	4.84	17^c^	Half-life = 64.4 min	300 mM 25 °C	(Kullartz and Pietruszka 2012)
*Haemophilus influenza*	23.6	7.5	40	0.14	70.42^b^	Maximal activity at 300 mM		(Woo et al. 2014)
*Staphylococcus epidermidis*	29.2	n.s.			67.1^c^	11.3% retention of activity	2 h 300 mM 25 °C	(Fei et al. 2015)
*Lactobacillus brevis* ECU8302	n.s.	6	40	3.34	102^b^	Half-life = 37.3 min	300 mM 25 °C	(Jiao et al. 2015)
Extremophiles (hyperthermophilic unless specified)								
*Aeropyrum pernix*	24.5	6.5	n.s.	0.057**	4.5^c^**	n.s.		(Sakuraba et al. 2003)
*Thermococcus kodakaraensis*	24.5	4	95	0.81***	285^b^***	n.s.		(Rashid et al. 2004)
*Pyrobaculum aerophilum*	24.5	6	n.s.	0.066	0.25^c^	53% retention of activity	20 h 300 mM 25 °C	(Sakuraba et al. 2007)
*Thermotoga maritima*	27.8	6.5	n.s.	0.02	1^c^	46% retention of activity	20 h 300 mM 25 °C	(Sakuraba et al. 2007)
*Hyperthermus butylicus*	26.4	5.5	80	0.15	0.5^c^	75% retention of activity	8 h 300 mM 25 °C	(Wang et al. 2010)
*Aciduliprofundum boonei*	26.6	7	80	0.12	n.s.	70% retention of activity	4 h 250 mM 25 °C	(Yin et al. 2011)
*Haloarcula japonica* (Halophilic)	26.6	6.4	60	1.02	8.92^b^	35% retention of activity	5 h 300 mM 25 °C	(Ohshida et al. 2016)

*n.s.* not specified

^a^Of monomer

^b^V_max_

^c^Specific activity

^d^One unit is defined as the amount of DERA required to cleave 1 μmol DR5P per minute

^e^Increase of activity till 65 °C; extreme loss in activity at 67 °C and 70 °C

*Measured at 37 °C

**Measured at 50 °C

***Measured at 95 °C

## Synthetic applications

### DERA in synthesis of natural product analogues

The formation of lactols such as **4** and **5** reported by Gijsen and Wong truly opened up avenues for DERA as a biocatalyst (Gijsen and Wong 1994). Wong et al. further used this sequential aldol reaction for the synthesis of epothilone fragments, in two separate reactions (Liu and Wong 2002). The enzyme successfully established stereocenters in the final molecule and proved to be a novel facilitator for highly complex anti-tumor agent epothilone A (**6**) (Scheme 5a).

### DERA in synthesis of pharmaceutical intermediates

An interesting application for the DERA-catalyzed sequential aldol reactions is in the production of HMG-CoA reductase inhibitors called statins. Statins are important cholesterol-lowering drugs and DERA provides a simple route to the common polyol motif in these drugs in a single step using achiral substrates like acetaldehyde (Gijsen and Wong 1994).

It is promising that several groups have pursued the synthesis of these statin precursors using DERA isolated from different organisms. Scientists at Diversa corporation developed a commercially attractive process by tackling two important factors, namely DERA activity and lactol productivity. This was done by identifying DERAs with superior features through environmental libraries and by using a fed-batch strategy to overcome enzyme inhibition by substrate (Greenberg et al. 2004). An industrial scale low temperature process for the same was also reported to have been developed by DSM, with a final product concentration of 100 gL^−1^ (Müller 2005). Ohshima and co-workers carried out sequential aldol reactions of acetaldehyde in the presence of DERA isolated from thermophilic organisms and concluded that even though these organisms were not as active as DERA_EC_, their performance in the synthesis was much higher, owing to the higher acetaldehyde resistance (Sakuraba et al. 2007). Shen and co-workers reported higher conversions when chloroacetaldehyde was used as the acceptor substrate, as compared to acetaldehyde (You et al. 2013), reaffirming earlier findings of Gijsen and Wong (Gijsen and Wong 1994, 1995a, 1995b; Gijsen et al. 1996). This was explained using the lower energy of chloroacetaldehyde for docking in the acceptor binding pocket from molecular docking studies. Using a similar argument, it was predicted that 3-azidopropionaldehyde would perform best in this reaction.

Sequential aldol addition of 3-azidopropanal to acetaldehyde gives a deoxy-azidoethyl pyranose which was oxidized to lactone **8**. Both, the nitrile **12** or the azido compound **8** can be used as a precursor for synthesis of the side chain of atorvastatin, rosuvastatin, and compactin. Also, many versatile pyranoid building blocks such as compound **11** can be prepared in large scale using the same strategy, a remarkable elimination followed by a subsequent Michael addition under retention of stereochemistry (Scheme 5b) (Müller 2005, 2012, Wolberg et al. 2008). A whole-cell approach to the statin side chain from chloroacetaldehyde or acetyloxyacetaldehyde and acetaldehyde was recently studied paying particular detail to all the relevant equilibria and pointing to its future potential (Oslaj et al. 2013).

### DERA-catalyzed preparation of deoxysugars

**Scheme 6:**
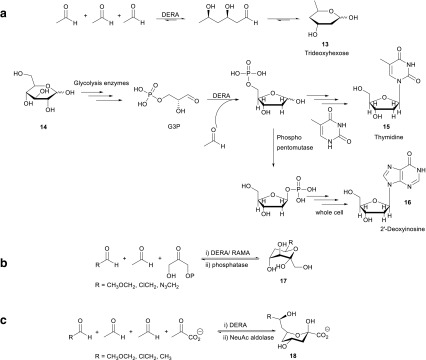
**a** DERA as a catalyst for the preparation of deoxysugars and thymidine or 2′-deoxyinosine. **b** Coupled DERA-RAMA-catalyzed sequential aldol reactions. **c** Coupled DERA-NeuAc-catalyzed sequential aldol reactions

DERA has been very successfully used in the preparation of different types of deoxysugars, such as deoxy, dideoxy, trideoxy, aza, and thio sugars. If acetaldehyde is the only substrate, it will react with itself and (3*R*,5*R*)-2,4,6-trideoxyhexose **13** was obtained as a product for self-aldol and cross-aldol reaction (Barbas et al. 1990; Gijsen and Wong 1994). In a recent study on the synthesis of 1,3-butadiol, the enzymatic reduction of the initial aldol product of two acetaldehydes stood central. The combination of the two enzymes has, however, not yet been investigated (Kim et al. 2017). DERA also provides a straightforward route towards pyrimidine nucleosides (Valino et al. 2012, 2015). Valino et al. obtained promising nucleoside **15** yields from cheap starting material like glucose, using a whole-cell cascade reaction involving DERA (Scheme 6). Earlier, a whole cell catalyzed route to 2′-deoxy inosine **16** had been developed, in which DERA played a key role (Scheme 6a) (Ogawa et al. 2003; Horinouchi et al. 2006).

### DERA-catalyzed preparation of deoxy-ketoses and deoxy-sialic acid

The one pot sequential reaction methodology can also be applied (Gijsen and Wong 1995a, 1995b) also using the combination of two aldolases. Combining the aldehyde formed after the DERA-catalyzed aldol reaction might undergo a second aldol reaction with DHAP catalyzed by RAMA to give a 5-deoxy ketoses **17**. Also, the combination of DERA with NeuAc aldolase gives several nine-carbon sialic acid-type sugar derivatives **18** (Scheme 6b, c). This remarkable triple aldol reaction is achieved by two sequential DERA-catalyzed aldol reactions, followed by the NeuAc catalyzed reaction with pyruvate.

## Acetaldehyde resistance of DERA

Even though DERA seems to be a promising tool for the industrial synthesis of chiral chemicals and is applied in the statin synthesis by DSM and Diversa, there is limitation in its uses for economically efficient large-scale synthesis as discussed earlier. Therefore, different groups have tried to tackle these issues and improve the properties of this enzyme.

### Genetic modification

Jennewein et al. employed directed evolution strategies combined with high throughput screening to identify mutations that have a positive impact on the chloroacetaldehyde resistance and catalytic activity of DERA. The most productive mutations were combined using site-directed mutagenesis to obtain a DERA variant that exhibited the desired properties (Jennewein et al. 2006). They found that the amino acid residues Phe200, Ile166, and Met185 form a hydrophobic cluster close to the active site Lys167 and Lys201 of DERA. On combining the successful point mutations in DERA_EC_, the authors obtained a tenfold improved variant to synthesis (3*R*,5*S*)-6-chloro-2,4,6-trideoxyhexapyranoside under industrially relevant conditions.

**Fig. 1:**
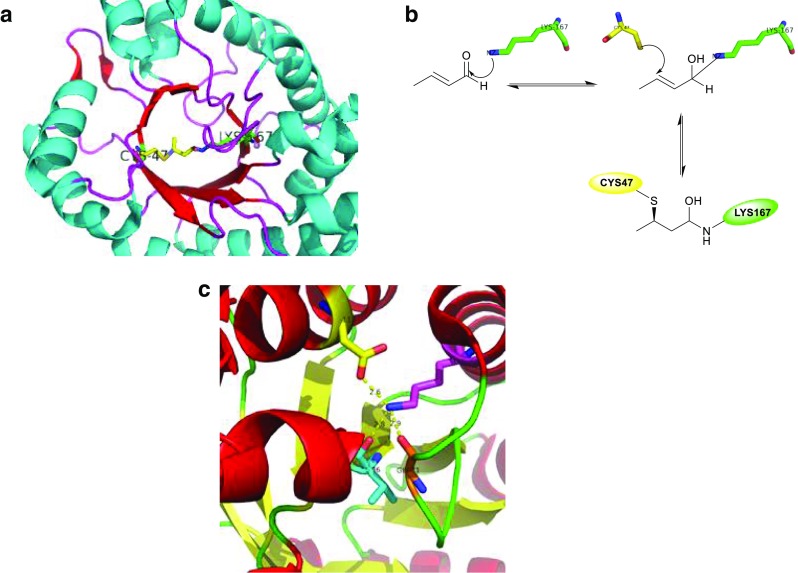
**a** Crystal structure of monomerized *E. coli* DERA shows aldol product bridging the catalytically active lysine Lys167 to a nearby cysteine Cys47 (PDB ID: 5EL1). **b** Proposed reaction mechanism showing the enzyme deactivation. **c** The crystal structure of DERA_LB_ Glu78Lys (PDB ID: 4XBS). The mutant Lys78 forms hydrogen bonds with Gly71 and Val96 at distances of 2.8 and 2.9 Å, and one salt bridge with Asp113 at a distance of 2.6 Å

Virtual mutation technology was employed along with site-directed mutagenesis to affect the rigidity of the protein structure and thus improve the acetaldehyde stability of DERA cloned from *Staphylococcus epidermidis* (SEP). Two of the variants in this study showed higher acetaldehyde resistance as compared to the wildtype and retained around 30% of initial activity after 24 h of incubation in 300 mM acetaldehyde. A variant with three independent point mutations (at positions 120, 174, and 213) also showed some increased tolerance, but the mutant showed a 50% reduction in initial activity (Fei et al. 2014, 2015). Dick et al. identified the product of aldol condensation between two acetaldehyde molecules acting as an inhibitor of DERA. A mechanism was proposed by the authors to explain this inhibition, where the product crotonaldehyde forms a Schiff base with the lysine side chain, followed by Michael addition of the cysteine thiol group to the C_β_ atom of the inhibitor (Fig. 1). There is no way to exchange the catalytic residue Lys167 without losing the enzyme function. Thus the substitution of cysteine with non-nucleophilic amino acid as methionine was identified as the best choice to give rise to an acetaldehyde resistant enzyme. The Cys47Met variant remains the most aldehyde resistant DERA_EC_ variant reported to date (Dick et al. 2016b).

However, it is important to note that follow-up studies where Cys47 was replaced with several other non-nucleophilic amino acids always yielded variants that were resistant to crotonaldehyde but failed to always give rise to acetaldehyde resistant DERAs. It was concluded that the acetaldehyde resistance of the enzyme was most likely correlated to the volume of the active site, with only one exception identified among the tested substitutions. Hence, it is possible that there might be another mechanism through which DERA is inactivated in the presence of acetaldehyde (Bramski et al. 2017).

In another study, it was aimed to expand the substrate scope and stereoselectivity of DERA for the synthesis of deoxy-azidoethyl pyranose which can be used as a precursor to cholesterol-lowering agent Lipitor. The Ser238Asp variant of DERA_EC_ showed a 2.5-fold improvement in activity towards the non-natural and toxic substrate 3–azidopropinaldehyde. A novel azidopyranose was formed in a sequential aldol reaction with the Ser238Asp mutant, whereas the wild-type enzyme was inactive towards the azidoaldehyde. DeSantis et al. suggested that this increase in the activity towards the non-phosphorylated substrate stems from the ability of the terminal hydroxyl group of the product to form a hydrogen bond with the carboxylate of Asp238 (DeSantis et al. 2003). In addition, this mutant also exhibited better performance in the condensation between 3-chloropropionaldehyde and acetaldehyde, with yields of 43% compared to the wildtype which gave 25% (Liu et al. 2004). In a recent study, these successful mutations reported for DERA_EC_ (Ser238Asp, Phe200Ile, Tyr259) were implemented on DERA from *K. pneumoniae* and a synergistic effect was observed with the mutant showing a threefold improvement in activity and almost a twofold increase in tolerance towards non-phosphorylated substrates (Li et al. 2015).

Another mutation (Glu78Lys) was introduced in DERA identified from *Lactobacillus brevis* (LB), which showed outstanding activity and substrate tolerance for catalyzing the reaction at high concentrations of substrates to give (3*R*,5*S*)-6-chloro-2,4,6-trideoxyhexapyranoside (Jiao et al. 2015). This mutant displayed tolerance to substrate concentrations up to 700 mM chloroacetaldehyde and 1400 mM acetaldehyde. The improvement of the stability and acetaldehyde tolerability in DERA_LB_ was attributed to the formation of two hydrogen bonds between the side chain of Lys78 and Gly71 and Val96 at distances of 2.8 and 2.9 Å, respectively, as well as one salt bridge with Asp113 at a distance of 2.6 Å (Fig. 1c).

To summarize, several different genetic modifications proved to be useful in improving the aldehyde resistance of DERA. While the mutants, in general, showed improved catalytic activity, some mutations did have a negative impact on the catalytic activity. The different mutations addressed in the above section have all targeted different areas of the active site and produced variants that seem to provide a starting point towards optimizing the enzyme for industrial DERA-catalyzed biosynthesis. Combining different point mutations also proved to be successful, with the mutations acting synergistically to improve enzyme properties.

### Immobilization

Immobilization of enzymes is a common strategy to ease their recycling. At the same time, it is also utilized to improve their stability, an essential parameter for enzyme recycling (Hanefeld et al. 2009). In both covalent and non-covalent immobilization methods, use of the functional groups on the enzyme surface is made. Consequently, these functional groups are then shielded and less susceptible to reactions that could lead to deactivation. Almost three decades ago covalent immobilization via lysine amino groups on the surface was already employed to stabilize a lipase against acetaldehyde induced deactivation (Berger and Faber 1991).

This type of protective covalent linking of the enzyme via a tether to the carrier material was also applied to stabilize DERA against high acetaldehyde concentrations. Three examples were described with different carriers but always utilizing the amino group of the surface lysines (Wang et al. 2012; Fei et al. 2014; Reinicke et al. 2017). In all cases, the active immobilized DERA displayed improved, but not excellent activity or stability. When non-covalent immobilization techniques were used again the amino groups of surface lysine that are susceptible to acetaldehyde are protected. When an acidic carbon nanotube was used as anionic carrier for ionic binding again, the lysine groups on the surface of the enzyme acting as counter ions are shielded from the acetaldehyde and indeed some improvement in stability was observed (Subrizi et al. 2014). This was also the case in pure physical adsorption on porous silica nanoparticles. Again, part of the enzyme surface is protected by the carrier (Nara et al. 2011). A recent structural study demonstrated that immobilization via the C-terminal his tag is impossible as the C-terminus is folded into the active site. At the same time, this also explained why his tag purification provided limited success (Schulte et al. 2018).

The to date most successful approach again is based on covalent immobilization, but in this case via the thiol groups of the four surface cysteines. These can react with suitable polymers and the DERA is then incorporated into thin films that display good activity and stability (Zhang et al. 2018).

While immobilization uses an external support to improve enzyme performance, another valuable strategy is to utilize natural barriers to protect enzymes from harmful substrates. This is done by carrying out whole-cell biocatalysis, where the cell itself acts as a barrier against the harmful acetaldehyde (Ishige et al. 2005). Valino et al. carried out hierarchical screening of different bacteria to identify strains with active DERA. *Erwinia caratovora* (*Pectobacterium atrosepticum*) was reported to give satisfactory yields of DR5P at all starting concentrations of acetaldehyde, which meant that the DERA from this variant was not deactivated in the presence of the aldehyde (Valino et al. 2012).

## Conclusion and outlook

The application of enzymes in the (stereo) selective synthesis of C–C bonds is well known. Among them, 2-deoxy-d-ribose-5-phosphate aldolase (DERA) is an interesting aldolase that catalyzes aldol reactions between two aldehydes. It is the only aldolase next to FSA that has been reported to accept both aldehydes and ketones as donors in an aldol reaction. This combined with the fact that it can also catalyze sequential aldol reactions to yield various precursors of biochemical and pharmaceutical importance ensures that DERA occupies a unique position among aldolases. However, the road to realizing large-scale biosynthesis with the enzyme has proven to be difficult.

Apart from the statin side chain industrial processes, there have been no recent reports of any commercial processes using the enzyme. A major reason for this is the fact that the enzyme is inactivated in the presence of acetaldehyde. Apart from this, catalyst loading and reaction times required to produce industrially relevant quantities of product are still quite high.

Protein engineering has contributed significantly to tackle the problem of aldehyde resistance in DERA by influencing key residues in the active site or by influencing the rigidity of the protein. However, the positive results reported by using different strategies do point to the fact that the exact mechanism of the inactivation is still not fully understood at this point. Hence, while we are in the right direction to realizing a commercial bio-synthetic route to statin precursors, there is still a long way to go before it is fully successful. In the future, more DERAs will be explored, further crystal structures of DERAs will be determined, and powerful computational technology will be used. First results are already known, such as the attempts to switch the enantioselectivity of DERA (Bisterfeld et al. 2016). While promising, further steps are necessary; given the large number of DERA structures and sequences known, this might also involve a shuffling-based approach (Stemmer 1994) which has so far not been attempted.

Moreover, mere protein engineering might not provide answers. For better chances of success, it is necessary to take a two-pronged approach to the problem. Protein engineering would need to be pursued along with diligent reactor engineering and enzyme immobilization approaches in order to achieve high activities and space-time yields along with lower rates of deactivation and reaction times. Thus, the industrial production value of DERA enzymes may be substantially increased by using computational design, immobilization, protein engineering, and rational reactor design.
